# Biological Determinants of Track and Field Throwing Performance

**DOI:** 10.3390/jfmk6020040

**Published:** 2021-05-07

**Authors:** Nikolaos Zaras, Angeliki-Nikoletta Stasinaki, Gerasimos Terzis

**Affiliations:** 1 Human Performance Laboratory, Department of Life and Health Sciences, University of Nicosia, Nicosia 1700, Cyprus; 2 Sports Performance Laboratory, School of Physical Education and Sport Science, University of Athens, 17237 Athens, Greece; agstasin@phed.uoa.gr (A.-N.S.); gterzis@phed.uoa.gr (G.T.)

**Keywords:** strength-power, lean mass, muscle fiber types, electromyography, muscle architecture

## Abstract

Track and field throwing performance is determined by a number of biomechanical and biological factors which are affected by long-term training. Although much of the research has focused on the role of biomechanical factors on track and field throwing performance, only a small body of scientific literature has focused on the connection of biological factors with competitive track and field throwing performance. The aim of this review was to accumulate and present the current literature connecting the performance in track and field throwing events with specific biological factors, including the anthropometric characteristics, the body composition, the neural activation, the fiber type composition and the muscle architecture characteristics. While there is little published information to develop statistical results, the results from the current review suggest that major biological determinants of track and field throwing performance are the size of lean body mass, the neural activation of the protagonist muscles during the throw and the percentage of type II muscle fiber cross-sectional area. Long-term training may enhance these biological factors and possibly lead to a higher track and field throwing performance. Consequently, coaches and athletes should aim at monitoring and enhancing these parameters in order to increase track and field throwing performance.

## 1. Introduction

Track and field throwing events are the shot-put, the discus, the javelin and the hammer throw. Performance in these events requires a well-developed movement technique and a high biological potential. In general, three major biomechanical factors affect throwing technique, and as a consequence, the distance of the throw: the angle of release, the height of release and the velocity of release [1,2,3,4]. Certainly, the velocity of release of the implement is the most important biomechanical parameter for achieving a high throwing performance [5,6,7,8]. Additionally, throwers are also distinguished for their large anthropometric characteristics, a prerequisite for a high-level throwing performance, and their well-developed muscle size, which is the result of inherited factors or the outcome from the systematic resistance training aiming to enhance strength and power [9,10,11]. Indeed, a large part of throwers’ training is dedicated to resistance training programs to enhance muscle strength, power and fast force production [12,13,14,15,16,17], and as a consequence, competitive track and field throwing performance.

From a practical perspective, the biological determinants which contribute to throwing performance may alter following long-term systematic training [18,19,20]. During the past decades, many studies have investigated the impact of these biological factors on competitive track and field throwing performance. However, several questions remain unanswered. For instance, track and field throwers are often characterized by large body sizes composed by large muscle mass and fat deposits, but the effect of excess body fat on throwing performance remains vague. Lean body mass is increased as a result of long-term training, with concomitant increases in maximum strength, power and rate of force development [20]. Yet, the absolute amount of lean body mass which would result in elite throwing performance, for each of the four throwing events, remains ambiguous. Also, it remains uncertain whether the association between lean body mass and throwing performance is linear even for athletes with large lean mass. Throwers are distinguished for their ability to generate large amounts of force on the throwing implements in short time-windows, usually between 150 and 240 milliseconds, generating high rates of force development [4,6,7]. However, the association between RFD of the lower and the upper extremities with throwing performance remains obscure. Additionally, the muscle fiber type composition and the architectural characteristics of the muscles which are thought to determine power performance are only scarcely explored in relation to track and field throwing performance [19,21,22]. For example, some studies revealed that elite throwers may possess a higher percentage of type II muscle fibers in their lower extremity musculature, while other studies did not reach the same conclusion. Therefore, it remains unknown whether a high percentage of type II muscle fibers is a criterion for elite throwing performance. Additionally, the muscle fascicle length seems to be linked with muscle power, but scarce data exist for throwers. Moreover, it remains unclear whether the activation sequence and intensity of the skeletal muscles during a maximal throw are linked with elite performance, although some data support such a premise [22]. Track and field coaches should be able to understand the training-induced changes in these biological factors and the possible link with performance in order to design more effective training programs and enhance track and field throwing performance. Therefore, the aim of this review was to accumulate and present the related literature regarding the role and the correlation of anthropometric characteristics, body composition, neural activation, muscle fiber type composition and muscle architecture characteristics with competitive track and field throwing performance. This information may assist athletes and coaches to design more effective training programs by means of a better understanding of the most important biological adaptations for throwing performance.

## 2. Article Selection Process

Manuscripts were searched manually with no limit regarding the year of publication. The literature review began in September 2020 and ended in November 2020. Only articles written in English were selected and further evaluated. Databases of PubMed, Google Scholar and Proquest were searched using the following keywords “shot-put throw”, “discus throw”, “hammer throw” and “javelin throw” in combination with the keywords “body mass”, “muscle mass”, “lean body mass”, “body fat”, “bone mineral density”, “EMG”, “neural drive”, “neural activation”, “rate of force development”, “muscle fibers”, “fiber type composition”, “muscle architecture characteristics”, “muscle thickness”, “fascicle length”, “fascicle angle”, “muscle adaptations”, “strength-power training” and “periodized training”. Furthermore, the keywords “track and field throwers”, “competitive track and field throwing performance”, “periodization” and “tapering” were also searched. Each article was evaluated for the participation of track and field throwers regardless of the level of performance, reporting at least one of the following biological determinants: anthropometric characteristics, body composition, neural activation, muscle fiber type composition and muscle architecture. All articles included in the initial database search were also examined for relevant articles in their reference lists. The analysis revealed 31 studies (from 1975 to 2021) related to the main question of this review [8,9,18,19,20,21,22,23,24,25,26,27,28,29,30,31,32,33,34,35,36,37,38,39,40,41,42,43,44,45,46].

## 3. Anthropometric Characteristics

### 3.1. Body Height

Body height directly affects one of the key biomechanical factors of throwing performance, the release height of the implement [32]. In addition, throwers should have a large arm-spread to maximize the range of force application on the implement [1,2,47]. Anthropometric data collected from athletes who participated in Olympic Games (1928, 1960–1976) showed that track and field throwers were among the tallest athletes of the Olympics, with the shot-put throwers being second in body height after the basketball players [26]. Data from Carter et al. [26] showed that discus throwers’ body height was approximately 1.88 m, hammer throwers’ was 1.83 m and javelin throwers’ was 1.82 m. Similar results became available from the 2009 IAAF World Championship in Athletics for discus throwers (1.98 m) and shot putters (1.93 m) [8,32]. Morrow et al. [27] also presented similar results: shot putters 1.87 m, discus throwers 1.92 m, hammer throwers 1.87 m and javelin throwers 1.83 m. These data suggest that a thrower must be tall enough to achieve a high throwing performance. Additionally, a study reporting on Indian shot putters showed that taller athletes (184.74 ± 3.45 m) may perform higher throwing distance than shorter athletes (180.00 ± 2.35 m) [36]. However, is there a relationship between body height and throwing performance? Recent data show that the correlation between body height and throwing performance is low and not significant in male shot putters (r = 0.18) and discus throwers (r = 0.24) [8,32], while the link between body height and javelin and hammer throw is unclear. This suggests that among track and field throwers of a similar level, body height is not the most decisive factor for performance, although being tall enough is necessary from a biomechanical perspective. In addition, the correlation between body height and competitive throwing performance in female throwers remains unexplored.

### 3.2. Body Mass

As expected, the large frame of track and field throwers corresponds to large body masses. Data from high-level thrower athletes show that, in general, throwers have body masses greater than 100 kg, except javelin throwers, who weigh less [26,27]. Contemporary data show that elite thrower athletes have become heavier, with the shot putters reaching 130 kg and the discus throwers approximately 117 kg of body mass [8,32]. Additionally, a study in Indian hammer throwers revealed that heavier throwers (90.00 ± 4.32 kg) were expected to perform a higher throwing performance compared to lighter hammer throwers (79.60 ± 3.78 kg) [37], while results from a study in sixty NCAA DI collegiate track and field athletes showed that throwers competing in shot-put, discus and hammer were heavier compared to jumpers, sprinters, mid-distance runners, pole vault athletes and javelin throwers [9]. Similarly, a study in male South Korean elite track and field athletes showed that throwers had greater body mass and strength compared to sprinters, jumpers and long-distance runners [48].

Body mass was not correlated with performance in shot-put performed either with the linear or the rotational technique, and discus throw [18,23,24]. Similar results have been presented for the hammer throw (r = 0.35) [33], although the authors of the current review are not aware of the relationship between body mass with javelin throw or with track and field throwing performance in female athletes. Unpublished data from our laboratory showed a low correlation between body mass and shot-put performance in 7 well-trained female athletes (r = 0.498) [44]. Body mass is the sum of lean body mass and fat mass. Resistance training increases lean body mass (mainly lean mass); however, body fat may vary greatly with altered nutritional habits, thus masking the link between body mass and performance. Therefore, a more intriguing question is whether there is a link between lean body mass and track and field throwing performance.

## 4. Body Composition 

### 4.1. Lean Body Mass

Lean body mass is considered one of the major biological parameters for strength/power performance and the rate of force development among thrower athletes [18,41,44]. Larger muscles produce greater muscle strength [49], and therefore greater muscle power. Accordingly, throwers regularly perform resistance training to increase their muscle strength and power, leading to a significant increase in lean body mass, especially in muscle groups directly involved in the specific throwing event. Thus, throwers possess higher lean mass compared to their track and field counterparts [9]. Table 1 presents the studies that have investigated the correlation between lean mass and track and field throwing performance. Shot-put performance with the linear technique is directly related to lean body mass estimated with skinfolds measurement [23,27]. Similar results were found for the relationship between lean body mass (evaluated with dual X-ray absorptiometry) and shot-put performance from the power position in novice throwers [30] and experienced shot putters [22,34]. Comparable to male linear shot-put throwers, recent evidence supports that total lean mass and trunk lean mass correlate with shot-put throwing performance in female shot putters using the linear technique [44].

Interestingly, lean mass was not correlated with rotational shot-put performance, especially during the competition period [34] in well-trained male shot putters. Additionally, it seems that the correlation between the percentage increase in performance and percentage increase in lean body mass after long-term training is low and not significant, which underpins the relatively small effect of chronic changes in lean body mass and rotational shot-put performance [34]. In concert with this, a recent case study showed that rotational shot-put performance varied independently of lean body mass in an elite male shot putter during a nine-year follow-up [38]. The rotational shot-put technique is a complex action requiring fast movement of the limbs during the power position and the final thrust compared to the linear technique [34]. It seems that factors other than the amount of muscle mass have a larger impact on rotational shot-put performance. Thus, the development of lean mass should not be the main objective for experienced male rotational shot-put throwers preparing for a competition, although a fairly large muscle growth should have been achieved before entering the competition period.

Nevertheless, a practical question is how much lean mass is necessary to achieve high-level throwing performance in shot-put. De Rose and Briazus [23] showed that shot-put performance with the linear technique above 19 m requires lean body mass greater than 115 kg. Morrow et al. [27] showed that performance above 17 m in the same event requires lean mass greater than 95 kg. However, these studies used skinfolds and equations for estimating body composition, which might have overestimated lean body mass. Recent studies with more advanced body composition analysis methodology (dual X-ray absorptiometry) estimated that 95 kg of lean mass is sufficient for 20 m shot-put performance with the rotational technique [38]. In addition, a study on Indian athletes showed that shot putters with greater lean mass may achieve higher performance compared to shot putters with lower lean mass [36]. Still, more research is needed in well-trained female shot-put athletes to determine the size of lean mass necessary for high shot-put performance, while a consensus on the method to evaluate total lean mass is needed to compare results from different studies.

Hammer throw is also a rotational throwing event but with higher strength and power demands compared to shot-put, while being more technically complex. Hammer throwing performance is closely correlated with total lean body mass, lower limbs lean mass and trunk lean mass [33]. These data, coming from well-trained hammer throwers (mean performance: 72.17 ± 6.40 m), suggest that one of the key training targets for hammer throwers should be the gain in muscle mass, mainly at the lower limbs and the body core. It has been calculated that for a performance above 75 m, a hammer thrower should have above 90 kg of total lean mass [33]. In contrast, two earlier studies failed to find any significant correlation between lean body mass estimated with skinfolds and hammer throwing performance in moderate level athletes [25,27], while a study on Indian athletes showed that hammer throwers with higher lean mass may achieve greater throwing performance compared to athletes with lower lean mass [37]. Hammer throw is highly correlated with backward overhead shot-put throw in well-trained hammer throwers (r = 0.95) [33]. Shot-put throws such as the backward overhead and the underhand are considered as whole-body throwing exercises, which are regularly used by all throwers during the year-round training [14,20,33]. Whittington et al. [31] presented a high correlation between lean mass and backward overhead throw in collegiate throwers (r = 0.81), while one more study verified this connection in a group of competitive male and female track and field throwers (r = 0.77 and 0.71) [41]. Thus, lean mass may be particularly important for strengthening the general throwing capacity in male and female athletes.

Scarce data exist for the relationship between lean body mass and discus and javelin throwing performance. Morrow et al. [27] showed a significant correlation between lean mass and discus performance, while it was calculated that discus throwing performance above 54 m in men requires 93.9 kg of lean mass. The same study failed to present any relationship with the javelin throwing performance, although it was calculated that javelin throwing performance above 65 m in men requires 82.9 kg of lean body mass. In addition, a recent study of our laboratory in well-trained track and field throwers, including two elite javelin throwers, showed that performance of 79.8 ± 1.8 m requires approximately 75.8 ± 2.8 kg of lean mass [43]. Discus and javelin throwing implements weigh less than the shot-put and hammer (2 kg and 800 g vs. 7.26 kg). Considering the force–velocity relationship, discus and javelin throws should depend more on the movement velocity and rate of force development than on maximum strength compared to the shot-put and hammer throw. However, accurate conclusions about the correlation between lean mass and discus and javelin performance both in male and female throwers need further investigation.

Altogether, it seems that lean body mass is closely related with linear shot-put and hammer throwing performance. For rotational shot-put, lean body mass seems to be of importance, but it may not be the key factor for high performance in rotational well-trained athletes, especially during the competitive period. In discus and javelin throws, lean body mass seems to be of lesser importance, although a certain, yet undefined, level of lean mass is the basis for excellent performance.

### 4.2. Bone Mineral Density

Another aspect of resistance training-induced increases in muscle mass is the resulting increased stress at the bone sites where muscles are attached. This compression or shearing stress induces bone adaptations, mainly the thickening of the bones and the increase in mineral density, to increase the bones’ ability to resist external loading [50]. Indeed, throwers have greater bone mineral density (BMD) than non-athletes and other athletes due to systematic resistance training [31]. Young competitive track and field throwers possess a total BMD of approximately 1.33 ± 0.08 to 1.35 ± 0.08 g·cm^−2^ [20,41], while well-trained shot putters and hammer throwers have total BMD of approximately 1.49 ± 0.01 and 1.48 ± 0.05 g·cm^−2^, respectively [33,34]. Thus, long-term systematic training, high load resistance exercises and increases in lean body mass may contribute to a higher BMD in well-trained throwers compared to young throwers and untrained individuals. Whittington et al. [31] showed that BMD is indirectly correlated with maximum isometric force (r = 0.68) and ball throw (r = 0.81) in collegiate throwers. However, BMD was not correlated with hammer throwing performance (r = 0.17, ns) in well-trained throwers, probably due to the small variation between athletes [33]. Still, the correlation between BMD and throwing performance in javelin and discus throw, as well as in all four throwing events in female athletes, needs further investigation.

### 4.3. Body Fat

Body fat may negatively contribute to power development. In fact, excess body fat may decrease movement velocity because of the extra body mass that needs to be carried by the muscular system. In this sense, the less body fat the better for a thrower. As an example, let us assume that there are two shot putters competing with the linear technique, with the same training background and physical/neuromuscular characteristics, except that the first has 10 kg and the second 20 kg of body fat. Let us further assume that the first athlete lands at the power position with 1.8 m·s^−1^ but the second athlete lands with 1.5 m·s^−1^ because his muscular system has to overcome 10 kg more mass (extra fat) during the glide. Assuming that both athletes will have the same velocity after taking the power position, the second one will have a release velocity handicap of 0.3 m·s^−1^, translating to 0.5–1 m of final throwing performance [51,52]. Along this line, Morrow et al. [27] and Whittington et al. [31] showed a significant negative relationship between body fat and hammer throwing performance (r = −0.79) and backward overhead ball throw (r = −0.89) respectively, although a recent study failed to show any correlation between body fat and performance in well-trained hammer throwers (r = 0.14) [33]. Studies showed diverse results about the percent body fat of throwers, perhaps due to diverse eating habits among athletes. Male throwers had an average body fat < 15–18%, while in female throwers, body fat may rise up to 25–28% [10,23,34]. Calculation of the data from Morrow et al. [27] revealed that the percent fat for shot putters was approximately 14.8%, for discus throwers 13.1%, for hammer throwers 15.3% and for javelin throwers 8.4%. Studies in Indian thrower athletes concluded that higher-level shot putters and hammer throwers possess less fat in comparison to lower-level throwers [36,37], while among NCAA DI collegiate track and field athletes, throwers possess the higher fat percentage compared to their track and field counterparts [9]. Unfortunately, the exact negative effect of excess body fat on throwing performance has not been calculated, especially in female athletes. However, throwers should adjust their nutrition habits to reduce their body fat to the levels that it will not interfere with their competitive performance.

## 5. Neural Activation

Regardless of the amount of muscle mass existing in a thrower’s body, this muscle mass must be activated in order to produce power. Accordingly, it is assumed that the recruitment of a large number of muscle fibers, especially type II muscle fibers, is necessary for high power outputs [53,54]. Muscle fiber recruitment during high-velocity movements is difficult to measure; therefore, researchers have attempted to evaluate the activation of muscles with surface electromyography (EMG). Recently, Howard et al. [42] evaluated the lower body muscle activation in 8 male (mean performance 11.50 ± 1.43 m) and 7 female (mean performance 11.53 ± 1.05 m) shot-put throwers using the glide technique. Results showed that the activation of rectus femoris and bicep femoris of the preferred leg as well as the activation of the bicep femoris of the non-preferred leg are crucial for shot-put performance in athletes with the glide technique. These results have significant practical applications in coaches and athletes to enhance the biomechanical requirements of the technique.

Scarce data exist regarding the relationship between muscle activation and throwing performance. Table 2 presents studies that have investigated the relationship between track and field throwing performance and EMG activation of the muscles. EMG amplitude of vastus lateralis and pectoralis major during a shot-put throw with the linear technique was closely correlated with the shot-put throwing distance in 8 well-trained shot putters [22]. This underpins the importance of the activation of these muscle groups during shot putting. However, it remains uncertain whether the higher EMG amplitude resulting in better performance is due to the recruitment of more muscle fibers or more type II muscle fibers. In the same study, it was also reported that shot-put throwing performance was negatively correlated with the duration between the activation of right vastus lateralis and right gastrocnemius muscle (r = 0.75). This finding further reinforces the technical directive of a fast movement velocity after landing to the power position.

**Figure 1:**
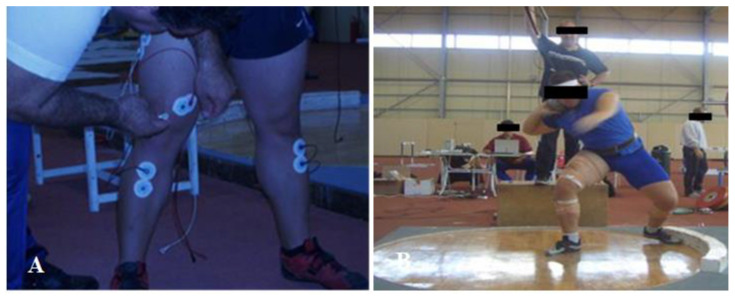
Electromyographic measurement of shot-put throw in well-trained shot putters using the rotational technique. (**A**) The placement of EMG on quadriceps muscles and (**B**) the measurement of shot-put throw with the rotational technique [18].

In a similar study, Kyriazis et al. [18] investigated the neuromuscular activation of vastus lateralis in shot-put athletes using the rotational technique, both at the beginning of the winter preparation phase and at the competition period, twelve weeks later (Figure 1). Vastus lateralis EMG was significantly correlated with shot-put throwing performance at both times, while vastus lateralis EMG muscle activation at the initial 200 ms was negatively correlated with shot-put performance, which reinforces the fast activation of the pushing leg on the power position during the full rotational technique. Additionally, vastus lateralis EMG was increased significantly following the 12-week training program (T1: 0.66 ± 0.23 mV vs. T2: 0.96 ± 0.44 mV), in parallel with the increase in shot-put performance (4.7% ± 2.0%) [18]. According to our knowledge, this is the only study that has investigated the effect of long-term training on vastus lateralis EMG in well-trained shot-put throwers. Consequently, twelve weeks of periodized training including throws, weightlifting derivatives, resistance training and plyometric exercises may increase the EMG activation and shot-put performance. The next interesting research/coaching question is to identify the appropriate training methods to increase neuromuscular activation during competition. Future studies in male and female throwers should examine the training-induced adaptations in EMG activation of protagonist muscles in order to establish the appropriate training methods to increase EMG activation and competitive throwing performance.

In discus throw, a strong correlation was reported between vastus lateralis EMG and performance from the power position and with the full rotational technique in well-trained discus throwers [35]. Additionally, a close negative relationship was found between discus throwing performance and the duration of the activation of quadriceps and gastrocnemius after taking the power position, which further supports the importance of fast activation of the lower limbs, especially during the full rotational technique. These correlations were found only for the muscles of the preferred leg, while no correlation was found between the non-preferred leg vastus lateralis and gastrocnemius muscles with discus performance. These results suggest that the action of left lower extremity muscles in right-handed discus throwers may not directly influence performance in discus. Similar to shot-put throw, coaches should focus on the fast activation of the right lower extremity during the power position of the full rotational technique, which may lead to faster discus velocity of release and a higher performance. Unfortunately, no data exists regarding the role of neuromuscular activation of the protagonist muscles in hammer and javelin throw in male athletes, while there is a lack of scientific data regarding the role of neuromuscular activation in female throwers.

Taken together, these results suggest that the EMG activation, especially of the lower musculature system, may correlate with competitive track and field throwing performance in shot-put (glide and rotational technique) and discus throw. Moreover, in shot-put, with the glide technique, the EMG signals of pectoral major significantly linked with shot-put performance. Throwers and coaches should focus on the fast activation of the lower-body musculature system, particularly when the athlete reaches the power position from the full technique in shot-put and discus throw.

## 6. Muscle Fibers

**Figure 2:**
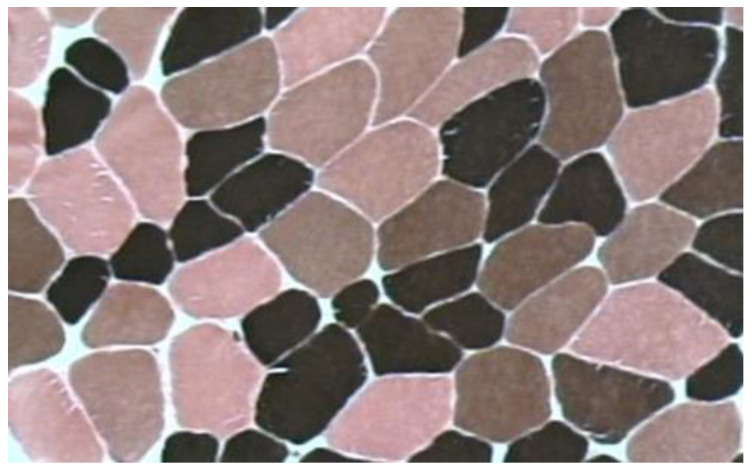
Myosin ATPase staining of a cross-section from vastus lateralis muscle of an elite hammer thrower (80.45 m best performance), preincubated at pH 4.6 and post-stained with eosin. Type I fibers appear as dark grey, type IIA fibers appear as moderate grey and type IIX fibers appear as light violet. Selective hypertrophy of type II fibers is obvious. Some small-sized type I fibers are shown [33].

There seems to be a consensus that fiber type composition and cross-sectional area (CSA) determine a large part of the muscle power capacity [53,55,56,57]. Three types of fibers have been identified in human skeletal muscles: type I, IIA and IIX, with type I having the lowest and type IIX the highest shortening velocities (Figure 2) [58]. There are several reports about the connection between power performance and type II muscle fibers [59,60]. Accordingly, an early study showed that shot putters with performance between 18.9 and 19.7 m and discus throwers (performance range: 60.9–61.3 m) had approximately 62.3% of type II muscle fibers in their lateral head of the gastrocnemius [21]. Javelin throwers performing between 76.2 and 81.1 m (with the older type of implement) had approximately 49.6% type II muscle fiber in gastrocnemius [21]. Later, Coyle et al. [24] showed that shot putters with best performance ranging between 19.14 and 20.33 m had a mean of 62.2% type II muscle fibers in their gastrocnemius muscle. In well-trained hammer throwers with mean performance of 72.17 ± 6.40 m, the percentage of type II muscle fibers in vastus lateralis was approximately 60.1% [33]. Interestingly, the percentage area covered (%CSA) with type II fibers was very similar between these athletes (66.1% ± 4%). As a comparison, in the same study, the muscle area covered with type I, IIA and IIX fibers was 49.0%, 37.9% and 13.1% respectively, in physical education students. Collectively, these data show that athletes using the heavier throwing implements have a predominance of type II fibers in their protagonist muscles.

Studies that have investigated the role of muscle fiber type composition and the possible link with track and field throwing performance are presented in Table 3. Analysis of the original data of the study of Coyle et al. [24] revealed that the percentage of type II muscle fibers in gastrocnemius was not correlated with shot-put throwing performance. Likewise, a study in novice throwers showed that the percentage of type II muscle fibers in vastus lateralis was also not correlated with shot-put performance [30]. Additionally, there was a low correlation between hammer throwing performance and the percentage of type II muscle fibers (r = 0.41) [33]. Therefore, although throwers have more type II fibers in their protagonist muscles, the percentage of these fibers may not be correlated with performance.

An interesting finding in a study with well-trained hammer throwers was that throwing performance was significantly correlated with the cross-sectional area (CSA) of type I, type IIA and type IIX muscle fibers [33]. Likewise, the %CSA occupied by type II muscle fibers from the long head of triceps brachii correlated with shot-put throw in novice throwers, but this correlation coefficient was reduced to low and non-significant when a certain extreme performer was removed from the analysis [29]. It seems that the role of the fiber CSA and %CSA is very important, especially when considering the heavier implements, i.e., the hammer and shot-put. This issue was highlighted in a case study of a world champion shot putter (personal best performance 22.75 m) [28]. Analysis of vastus lateralis biopsy sample, a few weeks after announcing the end of his career, revealed an unexpected low percent of type II fibers (40%), while the percentage of type II fibers of his colleague shot putter (21.01 m performance) was 67%, in agreement with previous studies. However, the world champion’s type II fiber CSA was enormous: 10,265 ± 465 μm^2^, elevating the %CSA occupied by type II fibers to 66.6%. These data suggest that a decisive muscle morphological characteristic for performance with the heavier throwing implements might be the absolute (in cm^2^) total CSA covered with type II muscle fibers. Alternatively, an athlete with initially low percentage of type II muscle fibers may excel in track and field throws provided that he/she will enlarge the type II muscle fibers and finally attain >62% of muscle area covered with type II fibers in lower body muscles.

Muscle fiber type composition is thought to be determined mainly by hereditary factors [61], although this still remains debated. Nevertheless, a common finding is that resistance training induces a transformation of type IIX to IIA muscle fibers, while detraining leads to the opposite fiber type transformation [53,55]. This phenomenon might be of great importance when preparing for a track and field throwing competition. As an example, fourteen weeks of resistance training per se increased shot-put throwing performance and induced a type IIX to IIA muscle fiber transformation [30]. After 4 weeks of complete detraining, shot-put performance remained unaltered but muscle strength and lean body mass were reduced. One way to explain the unaltered throwing performance between the end of the training and after 4 weeks of detraining might be the significant transformation of type IIA to type IIX muscle fibers found in this study [30]. This might suggest that transformations in type IIA to IIX with reduced training (e.g., during tapering) might induce noteworthy changes in throwing performance. Moreover, a frequent finding is that power training results in a preservation of the type IIX percentage muscle fibers [39,62,63,64], while the combination of strength and power training tends to maintain the percentage of type IIX muscle fibers [40,65]. This might have significant applications for performance in power-based sports like track and field throwing events, during year-round athletic preparation, when strength training is often combined with power training [20]. In agreement with this concept, a relatively high percentage of type IIX muscle fibers was found in vastus lateralis of elite hammer throwers at the end of the winter preparation period, when strength training volume was maximized but power training was concurrently performed [33].

In conclusion, muscle fiber type composition may not be a decisive factor for high throwing performance compared to CSA and %CSA occupied by type II muscle fibers. Muscle fiber type composition and CSA are significantly affected by strength and power training; consequently, coaches should design specific training programs according to the training period. These results show that for a high throwing performance in shot-put and hammer throw, a percentage of muscle fiber type II > 60% and a %CSA of type II > 62% may lead to an elevated throwing performance. Still, more research is needed for javelin and discus throw in both male and female athletes to reach certain conclusions.

## 7. Muscle Architecture Characteristics

**Figure 3:**
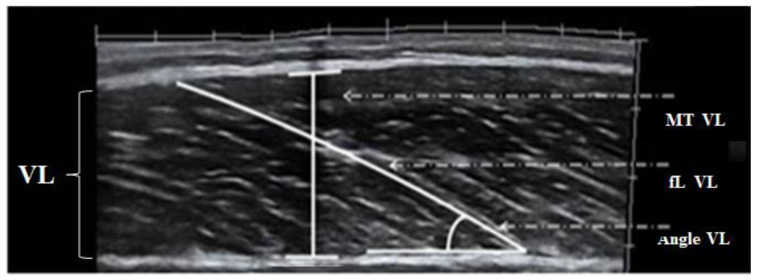
A comprehensive image of vastus lateralis (VL) muscle architecture including muscle thickness (MT), fascicle angle (Angle) and fascicle length (fL).

Muscle architecture characteristics, namely the muscle thickness, the fascicle angle and the fascicle length (Figure 3), have been considered as important factors contributing to muscle power production [66,67,68]. Muscle thickness and fascicle angle have been linked with muscle hypertrophy and strength [69,70]; thus, it is anticipated that athletes with greater muscle mass such as throwers may possess higher muscle thickness and fascicle angles. In addition, athletes with greater muscle thickness and fascicle angle may produce greater muscle strength and power compared to athletes with lower muscle thickness and fascicle angle [71]. In line with this, a study on well-trained throwers and taekwondo athletes showed that throwers possessed greater vastus lateralis muscle thickness (3.0 ± 0.5 cm vs. 2.4 ± 0.2 cm) and fascicle angle (22.7 ± 2.3° vs. 17.0 ± 2.5°) compared to taekwondo athletes. However, no significant difference was observed for vastus lateralis fascicle length (8.4 ± 0.8 cm vs. 8.4 ± 0.4 cm) [43]. Muscle fascicle length has been linked with fiber shortening velocity [72]. Studies showed that fascicle length was correlated with sprint performance [66,73], countermovement jump [43] and rate of force development [41,74] in well-trained power athletes and track and field throwers. However, only a handful of studies have examined the training-induced adaptations in muscle architecture characteristics in thrower athletes.

Generally, strength training may increase muscle thickness and fascicle angle, at least in previously untrained individuals [75,76,77]. In addition, Blazevich et al. [78] showed that only 5 weeks of resistance training is enough time to induce increases in muscle thickness, fascicle angle and fascicle length in trained participants. Nevertheless, during the past decade, a few studies examined the training-induced adaptations in muscle architecture characteristics and the possible link with throwing performance. Six weeks of strength training in novice throwers increased throwing performance (underhand, backward and front throw) by 7.0–13.5%, while vastus lateralis muscle thickness increased by 9.9% ± 2.6% [39]. Still, no significant changes occurred for fascicle angle and fascicle length. In the same study, six weeks of low-load, high-velocity ballistic-power training maintained the architectural characteristics of vastus lateralis, but throwing performance was significantly increased by 6.0–11.5% [39]. Similarly, 6 weeks of strength-power training with either compound or complex training methods in novice throwers led to increases in vastus lateralis muscle thickness (16.5% for compound vs. 7.1% for complex), vastus lateralis fascicle angle (26.1% for compound vs. 19.9% for complex) and gastrocnemius fascicle angle (5.3% for compound vs. 14.3% for complex), but significantly decreased the gastrocnemius fascicle length in the complex group by –11.8%. In addition, throwing performance increased only after compound training by 9.23% [40]. These studies investigated the role of muscle architecture in throwing performance but applied on novice throwers and used supplementary throwing exercises. Thus, a more interesting question is whether muscle architecture characteristics may affect competitive track and field throwing performance in thrower athletes.

In contrast to the above findings, different adaptations were found to experienced throwers (Table 4), mainly on fascicle length. Specifically, vastus lateralis muscle thickness and fascicle length increased either after a 12-week winter pre-competition training period (muscle thickness: 5.95% ± 7.13% and fascicle length: 13.4% ± 16.15%) or 10-week spring pre-competition training period (muscle thickness: 6.2% ± 7.4% and fascicle length: 10.5% ± 13.1%), but fascicle angle remained unaltered [41,45]. In line with these results, Bazyler et al. [19] presented that a 12-week training period designed with block periodization increased only vastus lateralis thickness (from 2.66 ± 0.45 cm to 2.84 ± 0.5 cm), but no changes were observed for fascicle angle or fascicle length. Tapering may also maintain the muscle architecture characteristics. More specifically, two weeks of tapering with either light or heavy loads maintained the vastus lateralis muscle thickness, fascicle angle and fascicle length in young competitive track and field throwers [20]. Consequently, muscle thickness increased in collegiate and young thrower athletes following systematic training, while two studies showed significant increases in fascicle length. However, a recent study in 12 well-trained track and field throwers (4 hammer throwers with performance range from 65.04 to 73.23 m, 4 javelin throwers with performance range from 64.91 to 79.72 m, 3 discus throwers with performance range from 50.55 to 55.84 m and 1 shot putter with performance of 15.52 m) showed that vastus lateralis muscle thickness and fascicle angle remained unaltered following 25 weeks of training, while fascicle length increased significantly [46]. Although changes in vastus lateralis fascicle angle were similar to previous studies, muscle thickness was higher compared to studies in collegiate (2.66–2.78 cm) [19] and young throwers (2.57–2.71 cm) [41,45], but similar with a recent study with elite weightlifters (2.97 ± 0.28 cm) [79]. In addition, a study in well-trained track and field throwers found similar results in vastus lateralis muscle thickness (3.0 ± 0.5 cm) [43]. Given the high level of the athletes that participated in the study, it might be speculated that vastus lateralis muscle thickness of approximately 3 cm may be dictated by the chronic adaptations to strength/power training in well-trained track and field throwers. However, such premise needs further investigation.

Competitive track and field throwing performance is partly correlated with muscle architecture. A study in young throwers showed that vastus lateralis fascicle length was significantly correlated with competitive track and field throwing performance, while a study in well-trained throwers showed that vastus lateralis muscle thickness was largely correlated with competitive track and field throwing performance [41,46] (Table 4). Furthermore, the linear combination of the percentage increase of vastus lateralis muscle thickness and fascicle length and the linear combination of the percentage increase of vastus lateralis fascicle length and the percentage change of fascicle angle explained approximately 33.8% and 33.5% respectively, of the percentage increase of competitive track and field throwing performance [41]. A great limitation of these studies was the participation of throwers from all four events (hammer, discus, shot-put and javelin). Therefore, it is not clear if this correlation between muscle architecture characteristics and throwing performance could be described for each event separately.

A possible answer for this question may provide the correlation between muscle architecture with shot-put throw from the power position and complementary shot-put throws (underhand and backward). Shot-put throw from the power position was correlated with vastus lateralis muscle thickness (r = 0.626) and fascicle length (r = 0.616 and 0.683) in young throwers [41]. In this concept, one more study in track and field throwers showed that shot-put throw from the power position was significantly correlated with vastus lateralis muscle thickness (r = 0.672) and fascicle length (r = 0.672), while the backward overhead throw was significantly correlated with vastus lateralis fascicle length (r = 0.895), following 10 weeks of training [45]. These significant but moderate correlations between shot-put throws and muscle architecture may not provide an analytical and comprehensive description about the connection of muscle architecture on throwing performance. Thus, future studies should focus in the most detailed investigation of this connection in well-trained male and female thrower athletes.

In summary, training-induced adaptations in muscle thickness, fascicle angle and fascicle length accompanied increases in strength, power and competitive track and field throwing performance. Long-term training may increase vastus lateralis fascicle length in track and field thrower athletes following the significant increases in competitive track and field throwing performance, even though there might be an upper threshold in vastus lateralis muscle thickness, especially in well-trained track and field throwers. Although a moderate correlation exists between muscle architecture and competitive track and field throwing performance, more research is required to strengthen the link between morphological adaptations and throwing performance.

## 8. Conclusions

The purpose of this review was to gather and present the existing data regarding the connection between biological factors (anthropometric characteristics, body composition, neural activation, fiber type composition and muscle architecture) and competitive track and field throwing performance. The main findings of the review are presented in Table 5. The current research evidence suggests that an elite thrower should be tall enough to increase the range of force application on the implement and increase the release height. Lean body mass is closely correlated with shot-put throwing performance with the linear technique both in male and female athletes, as well as with hammer throwing performance. Thus, one major training target in athletes competing in these throwing events should be the increase in muscle strength/mass, especially of the lower limbs. For shot putting with the rotational technique, increasing muscle mass may not be a main training target, as long as a minimum of approximately 85 kg of lean body mass has been established; in such case, power development and increased movement velocity should be the main training objectives. Excess body fat may interfere with throwing performance, and athletes should try to minimize body fat without affecting lean body mass. The neuromuscular activation of protagonist muscles such as the quadriceps and pectoralis major during a throw is a key factor for shot-put and discus performance, although the means to enhance this activation especially before competitions remains to be explored. In quadriceps muscle, a %CSA of more than 62% occupied by type II muscle fibers seems more likely to lead to elite throwing performance. This may be reached either with an increased inherited percent of type II muscle fibers and/or a training-induced increase of type II %CSA. Finally, the current data present that long-term training may increase vastus lateralis muscle thickness and fascicle length in track and field throwers, leading to a significant increase in competitive track and field throwing performance. However, the link between muscle architecture and competitive track and field throwing performance may be interpreted with caution because of the different throwing events of the athletes.

## Figures and Tables

**Table 1:** Correlations between lean body mass and track and field throwing performance in male and female thrower athletes.

Study	Athletes	Performance (m)	LBM Method	Total LBM (kg)	Correlation with Performance (Pearson’s r)
**De Rose and Briazus [23]**	5 Shot putters	16.72–19.28	Body diameters and skinfolds	102.8 ± 17.11	0.94 *
**Morrow et al. [27]**	13 Shot putters 9 Hammer 15 Discus 12 Javelin	17.57 ± 0.71 55.09 ± 4.77 53.56 ± 6.02 64.85 ± 4.97	Hydrostatic weighing, Siri equation	95.6 ± 5.9 88.3 ± 5.5 93.9 ± 6.9 82.9 ± 6.4	0.72 ** NS 0.55 * NS
**Terzis et al. [30]**	11 novice throwers	10.15 ± 1	DXA	62.7 ± 2.0	0.85 **
**Kyriazis et al. [34]**	9 Shot putters (Rotational Technique)	Pre-competition 13.97 ± 0.3	DXA	85.4 ± 1.7	0.70 *
		Competition 14.34 ± 0.3		85.5 ± 1.7	0.55, NS
**Terzis et al. [33]**	6 Hammer	72.17 ± 6.4	DXA	85.9 ± 3.9	0.81 *
**Singh et al. [36]**	20 Shot putters	NR	Skinfolds	80.70 ± 6.01 HP 75.09 ± 5.12 LP	NR
**Singh et al. [37]**	20 Hammer	NR	Skinfolds	71.9 ± 2.98 HP 64.64 ± 2.59 LP	NR
**Terzis et al. [38]**	1 Shot putter (Rotation Technique)	20.36	DXA	84.0–92.0	NS
**Anousaki et al. [44]**	7 Shot putters (Females)	13.90 ± 1.96	DXA	55.9 ± 3.8	0.93 **

* *p* < 0.05, ** *p* < 0.01, NR = not reported, NS = not significant, HP = high performance, LP = low performance, LBM = lean body mass, DXA = dual X-ray absorptiometry.

**Table 2:** Correlations between muscle electromyographic activation and competitive track and field throwing performance in shot-put and discus throwers.

Study	Athletes	Performance (m)	Muscles	Correlation with Performance (Pearson’s r)
**Terzis et al. [22]**	8 male linear shot putters	Ranged between 15.15 and 18.63 m	VL, PEC, TRI and GAS	Shot-put performance was significantly correlated with VL-EMG by r = 0.91 **, as well as with PEC-EMG by r = 0.75 *. No significant correlation was found between shot-put throwing performance and TRI-EMG or GAS-EMG.
**Kyriazis et al. [18]**	9 male rotational shot putters	Pre-Season 15.26 ± 1.67	VL	Shot-put performance was significantly correlated with VL-EMG both during pre-season and competition periods by r = 0.81 * and 0.80 *, respectively. A significant negative correlation was found between VL-EMG during the initial 200 ms of muscle activation and shot-put performance by r = −0.75 *, during both pre-season and competition periods.
		Competition 15.98 ± 2.11		
**Karampatsos et al. [35]**	6 male discus throw athletes	49.64 ± 4.3	Quant and Gas	Quadriceps EMG was significantly correlated with both standing and rotational discus throwing performance by r = 0.80 * and 0.81 *, respectively. A significant negative correlation was found between the duration of EMG activation of right quadriceps and right gastrocnemius with discus performance by r = −0.94 ** and −0.88 *, respectively

* *p* < 0.05, ** *p* < 0.01, VL = vastus lateralis, PEC = pectoral major, TRI = triceps brachii, Quant = quantriceps, GAS = gastrocnemius, EMG = electromyographic activity.

**Table 3:** Description of muscle fiber type characteristics in throwers and correlations between muscle fiber type composition, cross-sectional area and percentage cross-sectional area with track and field throwing performance.

Study	Athletes	Performance (m)	Muscle	Characteristics	Correlation with Performance (Pearson’s r)
**Costill et al. [21]**	3 male javelin throwers	78.6 (76.2–81.1)	LG	Type I = 50.4% (46.5–56.2) CSA Type I = 5585 mμ^2^ CSA Type II = 5771 mμ^2^ %CSA Type I = 47.7%	NS
	3 female javelin throwers	51.8 (49.1–57)		Type I = 41.6% (41.2–42) CSA Type I = 4864 mμ^2^ CSA Type II = 4562 mμ^2^%CSA Type I = 42.9%	
	4 male shot-put and discus throwers	61.1 (60.9–61.3) 19.3 (18.9–19.7)		Type I = 37.7% (13–52) CSA Type I = 7702 mμ^2^ CSA Type II = 9483 mμ^2^ %CSA Type I = 34%	
	2 female discus throwers	54.8 (53–56.6)		Type I = 51.2% (48.3–54) CSA Type I = 5192 mμ^2^ CSA Type II = 5851 mμ^2^ %CSA Type I = 46.9%	
**Coyle et al. [24]**	8 male shot putters	18.94 ± 0.26	LG	Type I = 37.8% ± 5.5% CSA Type I = 6367 ± 526 mμ^2^ CSA Type II = 6441 ± 749 mμ^2^	Type II (%) muscle fibers were poorly correlated with shot-put performance, r = 0.23, NS.
**Billeter et al. [28]**	1 male shot putter	22.75	VL	Type I = 60% Type II = 40% CSA Type I = 3430 ± 189 mμ^2^ CSA Type II = 10,265 ± 465 mμ^2^ %CSA Type I = 33.4% %CSA Type II = 66.6%	NR
**Terzis et al. [29]**	13 novice shot putters	10.90 ± 0.28	TRI	Type II = 64.6% ± 3.2% %CSA II = 71.4% ± 2.9%	Significant correlation was found between the %CSA of type II muscle fibers and shot-put performance (r = 0.70 *).
**Terzis et al. [33]**	6 male hammer throwers	72.17 ± 6.40	VL	Type I = 39.9% ± 5.0% Type IIA = 51.1% ± 9.0% Type IIX = 9.0 ± 7.0% CSA Type I = 5793 ± 670 mμ^2^ CSA Type IIA = 7703 ± 1171 mμ^2^ CSA Type IIX = 6554 ± 2040 mμ^2^ %CSA Type I = 33.9% ± 4.0% %CSA Type IIA = 57.3% ± 9.0% %CSA Type IIX = 8.8% ± 7.0%	CSA of Type I, Type IIA and Type IIX muscle fibers were significantly correlated with hammer throwing performance by r = 0.93 **, 0.96 ** and 0.90 **, respectively.

* *p* < 0.05, ** *p* < 0.001, NR = not reported, NS = not significant, LG = lateral head of gastrocnemius, VL = vastus lateralis, TRI = Triceps Brachii, CSA = cross-sectional area.

**Table 4:** Changes in muscle architecture characteristics following long-term periodized training and correlations between muscle architecture characteristics and track and field throwing performance in track and field throwers.

Study	Athletes	Training	Throwing Performance Change (%)	Muscle Characteristics	Correlation with Performance (Pearson’s r)
**Zaras et al. [20]**	2 Shot putters 4 Hammer 5 Discus 2 Javelin	13 throwers (7 males and 6 females) followed a year-round training macrocycle, leading into a 2-week tapering period with either light (30% of 1-RM) or heavy (85% of 1-RM) resistance loads.	LT: Throwing performance increased by 4.8% ± 1.0%.	VL-TH = 3.5% ± 6.4% ↔ VL-ANG = −4.4% ± 9.1% ↔ VL-LEN = 4.1% ± 10.2% ↔	NR
			HT: Throwing performance increased by 5.6% ± 0.9%.	VL-TH = 0.8% ± 4.1% ↔ VL-ANG = −1.7% ± 9.1% ↔ VL-LEN = 3.4% ± 9.0% ↔	
**Zaras et al. [41]**	2 Shot putters 4 Hammer 5 Discus 1 Javelin	12 throwers (6 males and 6 females) followed a 12-week periodized training program aiming to increase performance for the spring competitive period.	Throwing performance increased by 6.8% ± 4.3%.	VL-TH = 5.9% ± 7.1% ↑ VL-ANG = −2.5% ± 17.9% ↔ VL-LEN = 13.4% ± 16.2% ↑	Competitive track and field throwing performance (Z-scores) correlated with fascicle length only at T2 (r = 0.59 *). The percentage change of VL length and VL thickness tended to explain 33.8% of the percentage increase in track and field throwing performance (*p* = 0.09). Additionally, the percentage change of VL length and VL angle tended to explain 33.5% of the percentage increase in track and field throwing performance (*p* = 0.092).
**Bazyler et al. [19]**	3 Hammer 2 Discus 1 Javelin	6 collegiate track and field throwers (4 males and 2 females) followed 12-week training using a block periodization model culminating with a 1-week overreach followed by a 3-week taper. Here are presented the percentage differences for T1 to T3 measurements.	Throwing performance increased by 6.3%.	VL-TH: T1: 2.66 ± 0.45 cm T2: 2.84 ± 0.5 cm ↑ VL-ANG: T1: 21.74 ± 4.46° T2: 21.58 ± 4.23° ↔ VL-LEN: T1: 7.42 ± 2.06 cm T2: 7.85 ± 1.18 cm ↔	NR
**Zaras et al. [45]**	2 Shot putters 3 Hammer 5 Discus 1 Javelin	11 throwers (6 males and 5 females) completed 10 weeks of training aiming to increase track and field throwing performance for summer national competitions.	Throwing performance increased by 5.8% ± 2.8%.	VL-TH = 6.2% ± 7.4% ↑ VL-ANG = 1.4% ± 16.4% ↔ VL-LEN = 10.5% ± 13.1% ↑	Shot-put throw from the power position was significantly correlated with VL thickness before, r = 0.678 *, and after the training period, r = 0.669 *
**Anousaki et al. [46]**	1 Shot putter 4 Hammer 3 Discus 4 javelin	12 male well-trained throwers completed 25 weeks of training aiming to increase track and field throwing performance for the summer national competitions.	Throwing performance increased by 10.8%.	VL-TH = −1.5% ± 5.3% ↔ VL-ANG = −3.8% ± 11.5% ↔ VL-LEN = 9.6% ± 11.1% ↑	Competitive track and field throwing performance (Z-scores) was large to very large, correlated with VL muscle thickness at T1 (r = 0.547), T2 (r = 0.528) and T3 (r = 0.726 **).

* *p* < 0.05, ** *p* < 0.001, LT = light tapering, HT = heavy tapering, VL = vastus lateralis, TH = muscle thickness, ANG = fascicle angle, LEN = fascicle length, ↑ indicates significant increase, ↔ = indicates no significant change.

**Table 5:** Summary of the main findings of the review.

Anthropometric characteristics	Body height contributes to the height of release. Among track and field throwers of similar performance level, body height is less important for performance. Heavier throwers tend to achieve higher throwing performance compared to lighter throwers in shot-put and hammer throw, but the link between these two is weak.
Body composition	Lean body mass correlates with linear shot-put and hammer throwing performance. In rotational shot-put throw, lean mass is not correlated with performance in male athletes having >85 kg of lean body mass. Bone mineral density is not a good predictor for hammer throwing performance and general throwing performance. Body fat negatively correlates with hammer performance and backward overhead shot-put throw. A main goal for throwers should be the reduction of body fat.
Neural activation	Lower body musculature activation during the final thrust significantly correlates with shot-put and discus throwing performance. Upper body musculature activation significantly correlates with linear shot-put performance.
Muscle fiber type composition	Muscle fiber type composition of the lower body musculature may not be a decisive factor for elite throwing performance provided that >62% of the muscle cross-sectional area is covered with type II muscle fibers.
Muscle architecture	In well-trained throwers, vastus lateralis muscle thickness of ≥3 cm is related to higher track and field throwing performance. Fascicle length is moderately associated with competitive track and field throwing performance. Fascicle length may be increased with explosive and fast eccentric loading.
